# Distinguishing Doors and Floors on All Fours: Landmarks as Tools for Vertical Navigation Learning in Domestic Dogs (*Canis familiaris*)

**DOI:** 10.3390/ani14223316

**Published:** 2024-11-18

**Authors:** Lila Muscosky, Alexandra Horowitz

**Affiliations:** Dog Cognition Lab, Department of Psychology, Barnard College, New York, NY 10027, USA

**Keywords:** dog cognition, landmark learning, spatial navigation, vertical navigation, animal cognition

## Abstract

Spatial navigation allows animals to understand their environment position and is crucial to survival. An animal’s primary mode of spatial navigation (horizontal or vertical) is dependent on how they naturally move in space. Observations of the domestic dog (*Canis familiaris*) have shown that they, like other terrestrial animals, navigate poorly in vertical space. This deficit is visible in their use of multi-story buildings. To date, no research has been conducted to determine if dogs can learn how to navigate in an anthropogenic vertical environment with the help of a landmark. As such, we herein investigate the effect of the addition of a visual or olfactory landmark on dogs’ ability to identify when they are on their home floor. Subject behaviors toward their home door and a contrasting floor door were compared before and after exposure to a landmark outside of their home door. While subjects initially showed no difference in latency to approach an apartment door on their home or a wrong floor, we found a significant difference in latency to approach the doors in the test trials for subjects who approached the doors in every trial. Other findings are equivocal, but this result is consistent with the hypothesis that dogs can learn to navigate in vertical space.

## Simple Summary

Terrestrial animals, including humans and domestic dogs, naturally create cognitive maps in the horizontal plane in order to navigate; their navigation in the vertical plane is poorer, given their land-based locomotion. For humans, the development of multi-story buildings highlights this difficulty in mapping vertical space: for instance, on an elevator, humans typically rely on visual information about the height of the landing—floor numbers, e.g.,—to know where they are. However, human companions, domestic dogs, cannot rely on number cues, and do not appear to reliably know when the elevator has stopped on their home floor. In this study, we examined if dogs can learn what floor they are on based on a visual or odor cue on their floor, similar to humans’ use of a number cue. The subject dogs were able to do so, with some caveats. Our results suggest that cues may help dogs navigate an anthropocentric environment.

## 1. Introduction

Animals’ navigation of a complex environment—spatial navigation—can be considered along horizontal and vertical dimensions [1]. Preferred navigation style is typically dictated by the locomotion style of the animal: terrestrial animals are more skilled at horizontal navigation than at vertical navigation; animals who are both aerial and terrestrial can navigate in both horizontal and vertical dimensions.

When faced with the need to navigate on a vertical dimension, such as in multi-story buildings, humans still rely on horizontal spatial learning [2,3]. Similarly, anecdotal observations of domestic dogs (*Canis familiaris*) indicate that while dogs have good horizontal navigational skills, what evidence there is indicates they have a hard time understanding routes that have a vertical component [4]. In particular, it has been observed that when dogs are confronted with a non-natural vertical environment, such as a multiple-level apartment building, they do not appear to distinguish readily between different floors, especially when travel between floors involves the use of an elevator [5]. A preliminary study found that dogs in a novel multi-story environment were unable to identify which floor they were on [4].

Landmarks providing location information can enable learning, regardless of an animal’s navigation style [6]. Considering spatial navigation along the horizontal plane, previous research has shown that dogs are able to learn to use experimenter-provided visual landmarks to help find a novel object [7,8]. Other research has found that guide dogs’ shortcutting ability was improved after a single exposure to a new route, suggesting that wayfinding can be improved with minimal exposure [9].

In the present study, we attempted to determine if pet dogs could, similarly, use landmarks to, in effect, improve their vertical navigation skills. In particular, we tested whether landmarks would enable dogs to learn to distinguish different floors of their home apartment building and to identify when they are on the floor of their residence. Both visual and olfactory landmarks were used to gauge if the type of landmark influences dogs’ spatial learning. Previous studies have introduced visual landmarks to look at landmark learning in dogs [7,8,10]; cognition studies often fail to include an olfactory component in their experimental design, though dogs use olfaction as a primary sense [11]. In a human-designed world that is not organized around smell, dogs’ use of olfaction can be reduced, but the presence of novel odors has the potential to change their behavior [12]. As such, in this study, we aim to investigate the usefulness of both visual and olfactory landmarks to examine their effect on dogs’ spatial navigation.

We measured dogs’ approach and response to doors on two different floors in the building in which they lived with their owners: their own floor (“home” floor), both without and then with a landmark present, and an adjacent floor (the “wrong” floor). Either a visually novel item or a novel odor (in a minimally visible container) was presented, allowing us to gauge the salience of landmarks of varying modalities. We hypothesized that subject dogs would be able to use both kinds of landmarks to create a de facto vertical navigation skill, as seen in approach latency to the door on the home floor and the wrong floor of their building. In particular, we hypothesized that while subjects would initially not show a difference in latency to approach the wrong floor as the home floor, after exposure to a landmark on the home floor, they would show a difference, likely due to a decrease in approach speed to the wrong floor. We also anticipated that after exposure to the landmark, subject dogs would show more indirect approaches and exploratory behaviors on the wrong-door approaches. We expected that both landmarks would effect this change, but that the olfactory landmark would effect a greater change in subject behavior since dogs navigate through olfaction [11].

## 2. Materials and Methods

### 2.1. Participants

Domestic dogs and owners were recruited from the Barnard Dog Cognition Lab database. Twenty-nine dogs (12 M, 17 F) between the ages of 6 months and 12 years old participated in our study. Twelve dogs were described by their owners as purebred, and 17 dogs were mixed breed (Table 1). All participants resided in Manhattan, New York, and had lived in their present apartment building for at least four months at the time of the study.

Owners completed a preliminary questionnaire to narrow down possible participants based on apartment building regulations and layout. We gathered information about different building floor plans and about whether owners recognize the “wrong door” phenomenon in their dogs: the observation that, in many cases, dogs will approach the same door (same apartment line door) of a multi-story building from the elevator or stairs regardless of what floor they are on [4]. Selected participants confirmed that their dogs demonstrated the “wrong door” phenomenon.

Qualified participants were invited to fill out a second survey with details about their dogs and their living habits. Owners signed an electronic consent form before experimenters arrived at their apartment buildings for the study and were given information about their role in the study. Experimenters visited subjects’ apartment buildings on two occasions, approximately three to seven days apart (exact details outlined below). Testing took place in February and March 2024.

### 2.2. Experimental Procedure

Our experiment was designed to enable us to measure subjects’ approach and response to the doors on the wrong floor and their home floor before and after a novel landmark (outlined below) was placed outside their home door. Since subjects were familiar with their home floor and had established routines of going directly to that floor on returning to the building, all owners were asked to go to the wrong floor first. Apartment layouts varied, ranging from a short, straight hallway with only two apartment doors to a long hallway that turned corners with up to two dozen apartment doors. Subjects’ home floor number also varied from the second floor to the twenty-third floor.

#### 2.2.1. First Visit

Experimenters met the owner and subject outside their apartment building to explain the details of the study. Before the subjects entered the building, one experimenter went inside and placed a camera, a GoPro Hero 6, on a tripod in the hallway of the apartment building on the “wrong floor” in order to capture the subjects’ approach to the “wrong door”. The wrong floor was one floor upstairs from the subject’s home floor, or one floor downstairs if the subject lived on the top floor of the building. The wrong door was the door on the same line as their home door on the wrong floor: that is, the door just above or below the home door. The experimenter then moved out of sight so that their presence did not distract or affect the subject’s behavior. Owners were asked to approach the wrong floor first, rather than their home door, to avoid the unnaturalness of returning home and then immediately leaving. Owners thus took the elevator to the wrong floor and exited with their dog, as though they were going home. Owners were asked to let their dogs lead, or at minimum not to walk ahead of their dog. Once they reached the door, if they reached it, the recording was continued for 30 s. Then this trial was ended, and the owner returned to the lobby or ground floor of the building. The experimenter then moved the camera to the subject’s home floor, and the owner was cued to re-enter the elevator and proceed to their home door.

In this first visit, the videos served to capture the subject’s baseline behavior. After recording the baseline videos, the experimenter provided the owner with the landmark and demonstrated where to place it. Visual landmarks were placed just next to their home door, in the hallway, or on the home door; olfactory landmarks were attached to the door frame of the home door. Owners were instructed to draw the subject’s attention to the landmark’s presence, by tapping it, on every entrance to their home until the second visit. The second visit was scheduled in order to ensure the subject had ten or more encounters with the landmark [13], which took from three to seven days. Owners were asked to tally the number of times they entered and exited the apartment. The experimenters retrieved the camera and returned with the camera at the second visit.

#### 2.2.2. Second Visit

**Figure 1:**
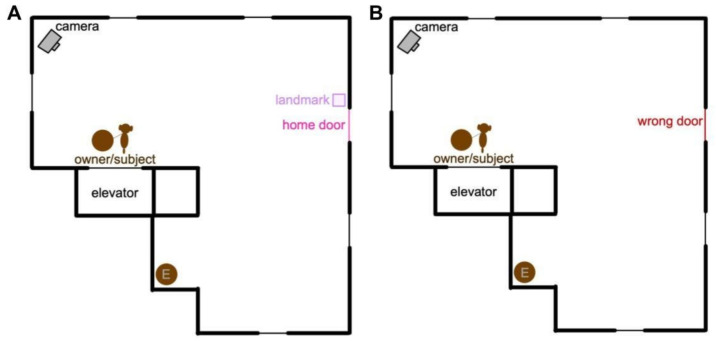
Example layout of an apartment building floor with the subject’s home floor (**A**) and a floor upstairs from the subject’s home floor, the wrong floor (**B**). In this example, the subject is presented with the visual landmark, the umbrella stand. The landmark is placed next to the subject’s home door. The camera is on a tripod and is situated to film the same door on both floors, out of the way from the subject. The experimenter (E) is hidden from view of the subject and owner.

Experimenters returned to the apartment building after subjects had at least ten encounters with the landmark (depending on the frequency of outings, the return was from three to seven days after the first visit) and placed the cameras in the same locations as before to keep consistency with the baseline videos. Except for the presence of the landmark, the procedure for the second visit otherwise matched the procedure for the first visit (Figure 1). Subjects were exposed to the landmark an average of 16 times (range 10–31) between the two visits.

### 2.3. Landmark

Subjects were randomly assigned to a condition with a visual or an olfactory landmark. Fifteen subjects were presented with a visual landmark. After pilot presentations to naive dogs not participating in the experiment, the default visual landmark chosen was a cuboid umbrella stand (5.9″ × 5.9″ × 16.9″); eleven subjects received this landmark, which was placed directly next to their home door. Due to apartment building regulations that restricted the placement of an object in the hallway, a second visual landmark was also used. For the alternative visual landmark, silver streamers were taped to the subjects’ home doors (*n* = 4 subjects).

Fourteen subjects received the olfactory landmark, a 365 Whole Foods^TM^ unscented dryer sheet inside of a small metal canister with holes in the lid. The “unscented” product was intended to be neutral to residents of the apartment buildings; at the same time, pilot dogs showed olfactory interest in the object (which does have an added scent). The olfactory landmark was placed inside of the door frame of the subject’s apartment door to minimize the landmark’s visibility.

### 2.4. Behavioral Coding

**Figure 2:**
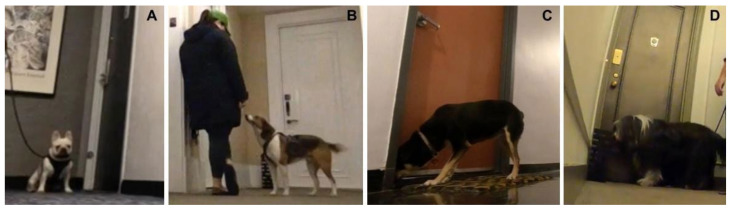
Examples of (**A**) approach, (**B**) look at owner, (**C**) sniff door, and (**D**) sniff landmark.

Video cameras captured subject behaviors after exiting the elevator or staircase at the home floor and the wrong floor for later frame-by-frame playback and coding. We developed an ethogram specifying behaviors indicating door approach, the approach latency from elevator to door, and various exploratory behaviors (Table 2). Approach was determined by whether the subject went toward the home or wrong apartment door, and latency measured the time in seconds that it took the subject to go from the elevator or staircase to the apartment door if they did approach. Trials ended after 120 s. Exploratory behaviors included sniff (the floor, door, or landmark), track, and look at owner (Figure 2). We compared the subjects’ behaviors on the first (baseline) and second (test) visit on each floor as well as between the home and wrong floors for each visit.

As these data are non-parametric, we performed statistical analysis using the Wilcoxon signed rank test to compare approach latency between the home and wrong floors in baseline and test trials and on the wrong floor between the baseline and test trials. The comparison is between the conditions since we could not control how often the owners had gone to a different floor in the past. We conducted these analyses twice: once including non-approaches, which were assigned the full trial length of 120 s, and once excluding non-approaches. A Chi-square test was used to compare the percentage of subjects to approach in the three contexts (*p* < 0.05).

## 3. Results

Of twenty-nine subjects, fifteen subjects approached all four doors in the trials; the rest only approached a subset of doors (*n* = 5 approached three doors; *n* = 5 approached two doors; *n* = 3 approached one door; *n* = 1 made no approaches). We primarily considered subjects who approached all doors, as a “non-approach” has no numerical equivalent so is not readily compared to latency figures statistically.

**Figure 3:**
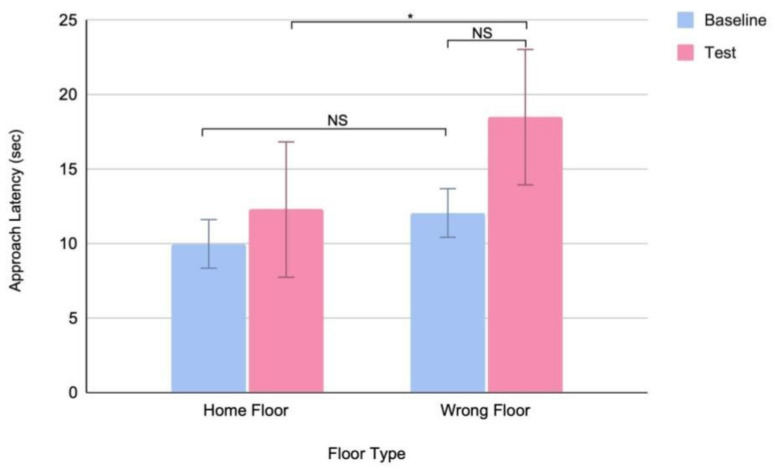
Mean (+/− SD) approach latency to the apartment door on the home and wrong floors of the building by dogs before (baseline) and after (test) they have time to learn a landmark placed outside of their door (* *p* = 0.03, NS: not significant).

There was no significant difference in latency to approach the doors between the home (mean = 9.98 s) and wrong (mean = 12.05 s) floor in the baseline trial (*n* = 18, Wilcoxon signed rank: Z = −0.74, *p* = 0.46) (Table 3). This suggests that dogs did not initially differentiate different floors of their building. We then compared the wrong floor (baseline versus test) and the test (home versus wrong floor) conditions to examine the effects of landmark placement, considering both the visual and olfactory landmarks. While there was no difference in latency to approach the wrong door between the baseline (mean = 12.05 s) and test (mean = 18.48 s) trials (*n* = 17, Wilcoxon signed rank: Z = −0.52, *p* = 0.60), there was a significant difference in latency to approach the doors between the home (mean = 12.28 s) and wrong (mean = 18.48 s) floor in the test trial (*n* = 18, Wilcoxon signed rank: Z = −2.16, *p* = 0.03), suggesting that subject behavior was influenced by the presence of a landmark (Figure 3).

**Figure 4:**
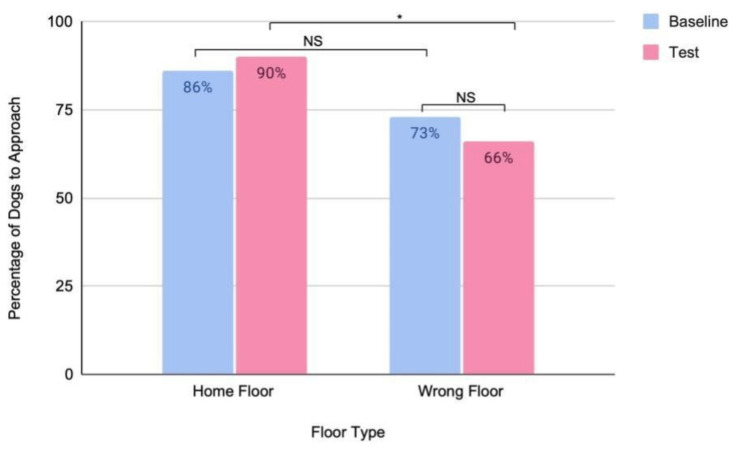
Percentage of dogs to approach the home and wrong floors of their building (*n* = 29) before (baseline) and after (test) they have time to learn a landmark placed outside of their door (* *p* = 0.03, NS: not significant).

While we were unable to compare latency in dogs who did not approach every door in a given trial, we are able to look at the approach rate. The approach rate increased on the home floor between the baseline and test trials (baseline = 86%; test = 90%), and it decreased on the wrong floor (baseline = 73%; test = 66%) between the baseline and test trials, suggesting some effect of the landmark placement. There was no significant difference in the number of approaches between the home (86%) and wrong (73%) floors in the baseline trial (Chi-square test: *p* = 0.31). There was a significant difference in number of approaches between the home (90%) and wrong floors (66%) in the test trial (Chi-square test: *p* = 0.03), further suggesting that the landmark helped subjects to differentiate between the two floors (Figure 4).

We also conducted pairwise comparisons including non-approaches in order to include the behavior of those subjects who did not approach one or more doors. A non-approach latency was considered to be the full trial length of 120 s. For each pairwise comparison, *n* varied based on the number of subjects who approached at least one door in the given comparison. With this in mind, there was a significant difference in latency to approach between the home (mean = 29.76 s) and wrong (mean = 49.71 s) floor in the baseline trial (*n* = 26, Wilcoxon signed rank: Z = −2.06, *p* = 0.04), which would indicate that dogs were initially able to distinguish between floors in their building. While considering non-approaches to be the trial ceiling of 120 s seems arbitrary, we performed this statistic as this strategy is used in some behavioral studies. Using this measure, latency to approach between the home (mean = 32.47 s) and wrong (mean = 53.43 s) floor in the test trial (*n* = 26, Wilcoxon signed rank: Z = −2.93, *p* = 0.003) differed, though latency to approach on the wrong floor between the baseline and test trials (*n* = 23, Wilcoxon signed rank: Z = −0.44, *p* = 0.66) did not.

### 3.1. Visual Versus Olfactory Landmark

All statistics for comparisons of subject responses to the type of landmark were completed including non-approaches to maximize the useable sample size for analysis. In the baseline trials, there was no significant difference in approach latency (between the home and wrong floor) for subjects later presented with a visual landmark (*n* = 14, Wilcoxon signed rank: Z = −1.1, *p* = 0.27), but there was a significant difference for subjects later presented with an olfactory landmark (n = 12, Wilcoxon signed rank: Z = −2.16, *p* = 0.03). In the test trials, there was no significant difference in approach latency (between the home and wrong floor) for subjects presented with the visual landmark (*n* = 14, Wilcoxon signed rank: Z = −1.70, *p* = 0.07), but there was a significant difference for subjects presented with the olfactory landmark (*n* = 12, Wilcoxon signed rank: Z = −2.73, *p* = 0.02). This suggests that the olfactory landmark may have contributed to subjects’ differentiation of different floors of their building, but, without comparing the trials directly, we cannot determine that.

There was no significant difference in the approach rate between the baseline and test trials on the home versus wrong floor for either the visual or olfactory landmark groups (visual home = 87%, visual test = 67%; olfactory home = 86%, olfactory test = 64%; visual and olfactory Chi-square test: *p* = 0.2).

### 3.2. Subject Behaviors on Baseline Trials (Pre-Landmark)

We examined subject behaviors to see if exploratory behaviors and indirect approaches differed on the wrong floor and on the home floor. There were insufficient subjects showing the behaviors to do any statistical analysis, but we include the data here descriptively. Most subjects had a straight approach to the door (home floor: *n* = 22; wrong floor: *n* = 15), but not all: on the wrong floor, seven subjects approached indirectly, and, on their home floor, three subjects approached indirectly. We looked at tracking, an exploratory behavior, and found 14 subjects showed tracking (wrong floor mean = 5.57 s, home floor mean = 4.95 s). Similarly, 15 subjects looked at their owner on the wrong floor with a mean latency of 5.51 s, and 21 subjects looked at their owner on their home floor with a mean latency of 8.77 s. Seven subjects moved away from the door on the wrong floor, while three moved away from their door on the home floor.

### 3.3. Subject Behaviors on Test Trials (Post-Landmark)

Of the subjects that approached in the test trials, most had a straight approach to the door (home floor: *n* = 15, wrong floor: *n* = 13), but not all: four subjects had an indirect approach on both the wrong and home floor. The number of subjects to show tracking behavior on both the home and wrong floors remained consistent from the baseline trial (home floor: *n* = 4, wrong floor: *n* = 11), but they were not the same subjects on both floors as from the baseline trials. The average tracking latency on the wrong floor was 6.1 s and on the home floor was 7.03 s. The number of subjects to look at their owner increased on both the home and wrong floors from the baseline trials (home: *n* = 23, wrong: *n* = 17), which includes the same 21 subjects from the baseline trial on the home floor, with an average latency of looking at their owner of 8.63 s on the home floor and of 6.24 s on the wrong floor. Additionally, on the wrong floor, 11 subjects moved away from the door, and six subjects moved away from the door on the right floor.

## 4. Discussion

In this study, owned domestic dogs learned how to navigate in the human-made environment—multi-story apartment buildings—with the help of a landmark. A premise of the study was that, consistent with Brandt et al. [2], subject latency to the “wrong door” (on a floor other than their home floor) was the same as their approach to the “right door” (their home). We first determined that dogs approached the wrong door on the wrong floor as quickly and often as they did the home door on the home floor. When considering complete trials, subject dogs in this study then learned to differentiate between their home floor and another floor of their apartment building after the introduction of a landmark. Thus, it appears that the landmark aided dogs in distinguishing between floors that they were previously unable to differentiate. This result is consistent with previous research on dogs’ ability to learn to associate a landmark with a novel object [8,10]. While we are unable to confirm if dogs are able to generate a true three-dimensional map, this result demonstrates a new use for landmark learning: in the context of vertical navigation, an unnatural form of navigation for terrestrial animals. While there are multiple cues in an elevator as to the floor at which it has arrived—including the length of time spent in an elevator, in a familiar building—it is notable that humans too rely on a visual “landmark” (the displayed floor number) to determine if they have arrived at the correct floor.

Among behavioral measures, we made a few notable, if very preliminary, observations. In looking at whether dogs approached the doors directly or indirectly, we found a decline in direct approaches during the test trial on the wrong floor, possibly indicating subjects’ recognition that they were on the wrong floor. A direct approach may indicate subject confidence in being on their floor as they exited the elevator and approached the door, while an indirect approach may signal the need to explore further. At the same time, against our main finding, the number of direct approaches on the home floor declined after the introduction of the landmark. This could be due to confusion induced by the dogs’ sequential approaches to apartment doors. We found longer bouts of tracking, an exploratory behavior [14], on the wrong floor than the home floor in the baseline trial, the reverse in the test trial, and more subjects tracking on the wrong floor in both trials. These observations do not lead to any definitive conclusion about subjects’ understanding; indeed, individual differences may account for their behavior. More subjects looked at their owner on the home floor in both trials, while more subjects moved away from the door on the wrong floor in both trials. These behaviors are equivocal: “looking at” could indicate that the subjects were looking for more information, or that they were waiting for their owners to act (by opening the door). Moving away from the door could suggest ambivalence, but it is not definitive. While the behaviors were meant to complement the latency measures, they are hard to interpret. And in all events, there was a small number of dogs showing these behaviors.

There are some caveats to our conclusions. A non-approach has no numerical equivalent, so it could not automatically be compared to latency figures. Including these subjects’ data create the problem that a non-approach needs to be quantified by a semi-arbitrary number if it is to be included in the analysis. We found that using a ceiling approach number skewed the data, with the analysis saying more about the particular number we chose than about subject behavior. However, including subjects who did not approach a door on a trial or two would have increased statistical power.

Due to restrictions in some residential apartment buildings, we were not able to leave our chosen visual landmark, an umbrella stand, outside the home door of some subjects and owners. Thus, we used a second visual landmark: streamers hung on the door. It is possible that one or the other object was less effective as a visual landmark for the subjects, thus diminishing the landmarks’ potency as a clue to their home floor. We used only one olfactory landmark, a dryer sheet cached in a canister at dog-nose level. When all subjects’ data were included, the olfactory landmark was more effective than the visual landmark. But with our smaller subject group of dogs who approached all doors on all trials, we were unable to properly compare the efficacy of these landmarks. Furthermore, while the olfactory landmark was designed to be inconspicuous, it was inevitably also visible and thus may have been noticed visually. Future studies might further examine the difference between visual and olfactory landmark presentation, as well as with a combined visual and olfactory stimulus.

It is important to note that latency may not be the only or best method to determine the salience and usefulness of the landmarks in approaching the doors. The advantage of this measure is its reliability: insofar as dogs were approaching the doors at all, the time to do so provided some indication of their recognition or impression of the environment. Other experimental methods, such as the cognitive bias test, use latency as the central measure [15]. On the other hand, the behavioral data, while equivocal in our case, are suggestive of the importance of individual differences that might, if captured, lead to a richer interpretation of subjects’ evaluation of their environment in the experimental context.

While this study provides evidence that the addition of a general landmark has an effect on approach behavior in dogs, future studies could more robustly examine the effect with a larger subject group. Additionally, while we made a practical decision to place a landmark close to the door, the design of some apartment buildings was such that some subjects could not see the landmark from the elevator. Thus, future work might look at a landmark placed at the elevator threshold (where the floor number is located): this may more powerfully indicate if dogs are disposed to use it in navigation. Finally, while our protocol asked owners to allow their dogs to lead them, rather than vice versa, this method was not consistent with how owners typically walked with their dogs. As with many experimental studies with owned dogs, owner behavior risks unduly influencing subject behavior; in this case, the reversal of the “leader” position in the dyad was unusual and may have affected some dogs’ behavior in an unspecified manner. Further, even if the dog was leading, we are unable to ensure that there were no signals between the dog and its owner that could interfere with our data.

Our results add to the literature about dog navigation, extending the work to consider navigation in a vertical plane. Examining dog behavior in an anthropogenic environment is important in helping to develop better methods for living with this ubiquitous and beloved animal companion.

## 5. Conclusions

Subject dogs demonstrate an ability to associate a learned landmark with their home floor in a multi-story apartment building. Thus, it appears that the landmark aided dogs in distinguishing between floors that they were previously unable to differentiate. This skill may be particularly useful in training service dogs who need to navigate vertically in addition to horizontally and has a potential application in improving pet dogs’ understanding of the human world as well. While preliminary, our results add to the inceptive literature about dog navigation, extending the work to consider navigation in a vertical plane.

## Figures and Tables

**Table 1:** Participants in the study. Information on subjects who participated in the study, their assigned landmark condition, and the number of exposures they had to the landmark. Landmark condition was either visual (U: umbrella stand or S: streamers) or olfactory.

Name	Age (yrs)	Breed	Sex	Landmark	Landmark Exposures
Aurora	3	Mixed	F	Visual (U)	22
Ava	8	Mixed	F	Visual (U)	16
Butterbean	3	French Bulldog	M	Olfactory	14
Cammy	5	Mixed	M	Olfactory	28
Charley	11	Miniature Poodle	F	Olfactory	11
Clue	2	German Shepherd	F	Olfactory	12
Daisy	2	Basset Hound	F	Olfactory	12
Donald	9	Mixed	M	Visual (U)	13
Eli	2	Mixed	M	Visual (U)	16
Holly	11	Mixed	F	Visual (U)	17
Iris	9	Airedale Terrier	F	Visual (S)	22
Janie	7	Beagle	F	Visual (S)	13
Koa Bean	4	Mixed	M	Olfactory	13
Lemmy	6	Cavapoo	M	Olfactory	13
Lucky	7	Mixed	F	Visual (S)	18
Maeve	2	Mixed	F	Olfactory	31
Mario	3	Maltipoo	M	Olfactory	14
Molly G	9	Cockapoo	F	Olfactory	21
Molly S	4	Mixed	F	Olfactory	19
Moxie	11	Mini Goldendoodle	F	Visual (U)	21
Nora	6 mo.	Samoyed	F	Visual (S)	8
Owen	2	Welsh Springer Spaniel	M	Visual (U)	10
Pedro	5	Mixed	M	Olfactory	15
Penny	10	Mixed	F	Visual (U)	11
Petey	6	Mixed	M	Visual (U)	21
Stella	10	Standard Poodle	F	Visual (U)	12
Wally	9	Bearded Collie	M	Visual (U)	18
Wednesday	2	Mixed	F	Olfactory	20
Zucchini	1	Pomeranian	M	Olfactory	14

**Table 2:** Ethogram used to code subjects’ behaviors on each floor of the building, with description of each behavior.

Behavior	Description
**Approach**	
Latency	Time (seconds) from elevator to door
Straight	Direct approach to door
Indirect	Indirect approach to door
Distraction	Person/item/sound diverts subject’s attention
Track	Sniffs floor while walking
Look at owner	Head directed toward the owner
**At Door (arm’s length)**	
Sniff floor	Nose is pointed at the base of the door or doormat
Sniff door/landmark	Nose is pointed towards the door or the landmark
Move away	Walks away from the door within 10 s of approach

**Table 3:** Results of analyses on latency to approach (Wilcoxon signed rank test).

Floor Comparison	Condition	Mean Latency	Test Statistic (*Z*)	*p*
Home vs. wrong	Baseline	Home: 9.98, Wrong: 12.05	−0.74	0.46
Home vs. wrong	Test	Home: 12.28, Wrong: 18.48	−2.16	0.03
Baseline vs. test	Wrong Floor	Baseline: 12.05, Test: 18.48	−0.52	0.60
Visual home vs. wrong	Baseline	Home: 13.03, Wrong: 14.44	−0.3	0.27
Visual home vs. wrong	Test	Home: 12.23, Wrong: 17.72	−1.36	0.07
Olfactory home vs. wrong	Baseline	Home: 6.92, Wrong: 9.66	−1.42	0.03
Olfactory home vs. wrong	Test	Home: 12.31, Wrong: 19.24	−1.72	0.02

## Data Availability

The data referenced in this manuscript will be made available by the authors, without undue reservation, to any qualified researcher upon request.
